# Expressed Sequence Tags for Bovine Muscle Satellite Cells, Myotube Formed-Cells and Adipocyte-Like Cells

**DOI:** 10.1371/journal.pone.0079780

**Published:** 2013-11-05

**Authors:** Eun Ju Lee, Majid Rasool Kamli, Smritee Pokharel, Adeel Malik, K. M. A. Tareq, Abdul Roouf Bhat, Hee-Bok Park, Yong Seok Lee, SangHoon Kim, Bohsuk Yang, Ki Young Chung, Inho Choi

**Affiliations:** 1 School of Biotechnology, Yeungnam University, Gyeongsan, Republic of Korea; 2 Bovine Genome Resources Bank, Yeungnam University, Gyeongsan, Republic of Korea; 3 Institute of Agriculture and Life Sciences, Gyeongsang National University, Jinju, Republic of Korea; 4 Department of Life Science and Biotechnology, College of Natural Sciences, Soonchunhyang University, Asan, Korea; 5 Department of Biology, Kyung Hee University, Seoul, Republic of Korea; 6 Hanwoo Experiment Station, National Institute of Animal Science, RDA, Pyeongchang, Seoul, Republic of Korea; University of Minnesota Medical School, United States of America

## Abstract

**Background:**

Muscle satellite cells (MSCs) represent a devoted stem cell population that is responsible for postnatal muscle growth and skeletal muscle regeneration. An important characteristic of MSCs is that they encompass multi potential mesenchymal stem cell activity and are able to differentiate into myocytes and adipocytes. To achieve a global view of the genes differentially expressed in MSCs, myotube formed-cells (MFCs) and adipocyte-like cells (ALCs), we performed large-scale EST sequencing of normalized cDNA libraries developed from bovine MSCs.

**Results:**

A total of 24,192 clones were assembled into 3,333 clusters, 5,517 singletons and 3,842contigs. Functional annotation of these unigenes revealed that a large portion of the differentially expressed genes are involved in cellular and signaling processes. Database for Annotation, Visualization and Integrated Discovery (DAVID) functional analysis of three subsets of highly expressed gene lists (MSC233, MFC258, and ALC248) highlighted some common and unique biological processes among MSC, MFC and ALC. Additionally, genes that may be specific to MSC, MFC and ALC are reported here, and the role of *dimethylarginine* *dimethylaminohydrolase2* (*DDAH2*) during myogenesis and *hemoglobin* *subunit* *alpha2* (*HBA2*) during transdifferentiation in C2C12 were assayed as a case study. *DDAH2* was up-regulated during myognesis and knockdown of *DDAH2* by siRNA significantly decreased myogenin (*MYOG*) expression corresponding with the slight change in cell morphology. In contrast, *HBA2* was up-regulated during ALC formation and resulted in decreased intracellular lipid accumulation and *CD36* mRNA expression upon knockdown assay.

**Conclusion:**

In this study, a large number of EST sequences were generated from the MSC, MFC and ALC. Overall, the collection of ESTs generated in this study provides a starting point for the identification of novel genes involved in MFC and ALC formation, which in turn offers a fundamental resource to enable better understanding of the mechanism of muscle differentiation and transdifferentiation.

## Introduction

Myoblasts and adipoblasts arise from the same mesoderm layer in embryos [1], and once formed, the cell population in adults is maintained by resident stem cells present at specific sites in the tissue. The multipotential capacity of resident muscle satellite cells (MSCs) to differentiate into myogenic, adipogenic and osteogenic cells has been extensively investigated [2,3]. MSCs have been differentiated into myotube-formed cells (MFCs) or transdifferentiated into adipocyte-like cells (ALCs) [4,5]. MFCs represent tubular structured cells with multiple nuclei resulting from proliferating myoblasts after they exit the cell cycle, differentiate and fuse. In contrast, ALCs are uni- or multi-nucleated myoblast cells with intracellular lipid forming capacity [6]. Transcription factors (myogenic - *Myf5, MyoD, MYOG, MRF*, and adipogenic - *CEBPα, PPARγ*), signal transduction complexes (Wnt and Notch), surrounding extracellular matrix environment (*M-cad*, *integrin*, *fibronectin*) and availability of oxygen [7] are the major determinants of the fate of MSCs [8]. Moreover, overexpression of regulatory markers such as *Myf5* has resulted in differentiation of other cells into myocytes [9], while ectopic overexpression of the adipogenic marker *PPARγ* has resulted in differentiation of myoblasts into adipocytes [10]. However, unlike muscle cell differentiation, studies of MSCs transdifferentiation into ALCs are limited and this process is still a matter of debate. Investigations of mouse [4,5,11] and human myoblasts [12] have been carried out to understand the basic mechanism involved in the switch towards ALC formation. 

We previously generated ESTs from a porcine normalized cDNA library and identified differentially expressed genes during adipogenesis [13]. Normalized cDNA libraries have a decreased prevalence of clones representing abundant transcripts, thus increasing the efficiency of random sequencing essential for new gene discovery [14]. Expressed sequence tags (EST) provide basic information for gene discovery, mapping, genetic variation and transcriptome analysis [15–17]. These ESTs serve as a structural and functional genomics tool for the identification of tissue specific marker genes, which in turn may aid to improve the meat quality and quantity in domestic animals [18,19].

Additionally, our earlier work on microarray analysis revealed a close relationship between gene expression profiles of different muscle and fat depots in bovine models [6]. However, the total number of probes used for the study only targeted transcripts of 16,341 genes, which covers less than 70% of the total number of genes in bovines [6]. Thus, for further identification of genes, validation of our microarray results, and to include the additional genes, normalized cDNA libraries from bovine MSCs, MFCs and ALCs were constructed. EST analysis of these bovine primary cells revealed the involvement of many genes during MFCs and ALCs formation, including some with unknown function. These approaches have led us to successfully identify genes like *TTR* (a thyroid hormone transporter in blood) from bovine skeletal muscle, whose functional role was elucidated in C2C12 cells during myogenesis [20]. Therefore, the ESTs generated in this study enabled us to identify several genes including *dimethylarginine dimethylaminohydrolase 2* (*DDAH2*) and *hemoglobin subunit* alpha *2* (*HBA2*) as well as their novel roles during differentiation and transdifferentiation respectively, in C2C12 cells. 

Overall, the current study forms a basic platform for the functional analysis of identified genes and further study of these known and unknown genes may provide insight into common pathways involved in myogenesis and adipogenesis. 

## Materials and Methods

### Cell culture and RNA extraction

Skeletal muscles from Korean native cattle aged 22-24 months with an average body weight of 550-600 kg were used for this experiment. All animals were handled according to a protocol approved by the Animal Care and Concern Committee of the National Institute of Animal Science, Korea. Briefly, the collected muscle was minced into fine pieces and digested with trypsin-EDTA (GIBCO, CA, USA), after which the samples were centrifuged at 90×g for 3 min and the upper phase was passed through a 40-μm cell strainer. The filtrate was then centrifuged at 2,500 rpm, after which the cell pellet was collected, washed twice and cultured in Dulbecco’s modified Eagle’s medium (DMEM; HyClone Laboratories, UT, USA) supplemented with 10% fetal bovine serum (HyClone Laboratories) and 1% penicillin/streptomycin at 37°C under 5% CO_2_. The emphasis was given on the primary MSC condition and the culture medium was changed every other day. Cells were treated with a transdifferentiation cocktail (TDC) at 70% confluency and allowed to grow for 7 days. To induce differentiation, cells were allowed to grow in DMEM without reducing serum (DMEM with 10% FBS and 1% P/S). A detailed description of MSCs isolation, differentiation, and transdifferentiation is described in our previous studies [6,21]. Cells were harvested for RNA isolation on day 10 of culture for MSCs and day 14 for MFCs. Similarly, MSCs switched to transdifferentiation media on day 10 were harvested on day 17 for ALC. Total RNA extraction and mRNA purification were conducted according to Lee et al. [11]. Briefly, equal amounts of RNA samples collected from cultured MSCs, MFCs and ALCs were pooled together, after which mRNA was purified from each pool of total RNA (100 μg) using the absolutely mRNA purification kit (Stratagene, CA, USA) and used for subsequent library construction. As for C2C12 cells, the cells cultured in 10% FBS media were switched to 2% FBS differentiation media when they reached 70% confluence. The cells were then harvested in Trizol, after which RNA was isolated and stored in DEPC water at -80°C until use. Total RNA extraction and cDNA synthesis were conducted as previously described [6].

### Normalized cDNA library construction

Normalized cDNA libraries from bovine primary cells were generated using the duplex-specific nuclease (DSN) based normalization method described by the manufacturer (Evrogen JSC, Russia). A directional Lambda ZAP cDNA Synthesis and Gigapack III Gold Cloning Kit (Stratagene, CA, USA) was used for construction of the cDNA library. Briefly, 5 μg of mRNA was reverse transcribed for first strand cDNA synthesis using an oligo-dT linker-primer containing a *Xho*I cloning site by incubating the samples at 42°C for 1 hour. To synthesize the second cDNA strand, RNase H (1.5 U/ μl) and DNA polymerase-I (9.0 U/μl) enzymes were added and synthesized for 2.5 hrs at 16°C. *EcoR*I linkers were then ligated into the 5´-termini of cDNA, after which normalization of the cDNA library was conducted using DSN as previously described [13].The products were then separated on a column containing Sepharose® CL-2B gel filtration medium to yield three fractions ranging in size from 500 bp to 1.5 kb, which were subsequently ligated into ZAP Express vector (pBK-CMV) to produce primary libraries of MSCs, MFCs, and ALCs. *In vitro* packaging of the ligation product was conducted using a ZAP Express cDNA Gigapack III Gold Cloning Kit (Stratagene, CA, USA). Excision cloned fragments were packed in phagemids and infected with *E.coli* strain XLOLR. The *E. coli* were then plated on LB-Kanamycin (50 μg/ml) containing X-gal/IPTG for blue/white selection. White colonies were randomly and manually selected and inoculated into a 384-well plate (Corning, NY, USA) containing 40 μl TB with Kanamycin (50 μg/ml), after which the samples were then incubated for 16 hours at 37°C, mixed with glycerol solution (65% glycerin, 0.1M MgSO_4_, 0.025M Tris-HCl, pH 8.0) and stored at -80 °C. Insert sizes were confirmed by PCR. 

### DNA sequencing

Sequencing of bovine MSCs, MFCs, and ALCs cDNA clones was performed as previously described [13]. Briefly, single plasmid colonies were cultured in terrific broth (TB) medium supplemented with Kanamycin (50 μg/ml) and then plasmid was purified using alkaline lysis method [22,23]. Sequencing reactions were performed using 250 ng plasmid DNA as a template with the3 pmol T3 primer (5'-ATTAACCCTCACTAAAG-3') and Big Dye Terminator v3.1 using a GeneAmp PCR System 9700 (Applied Biosystems, CA, USA). PCR products were purified by ethanol precipitation and DNA sequences were obtained using an ABI 3730 XL DNA Analyzer (Applied Biosystems). The nucleotide sequences obtained in this study have been submitted and are available in DNA Data Bank of Japan (DDBJ) under accession number HX915285- HX939203.

### EST analysis

**Figure 1:**
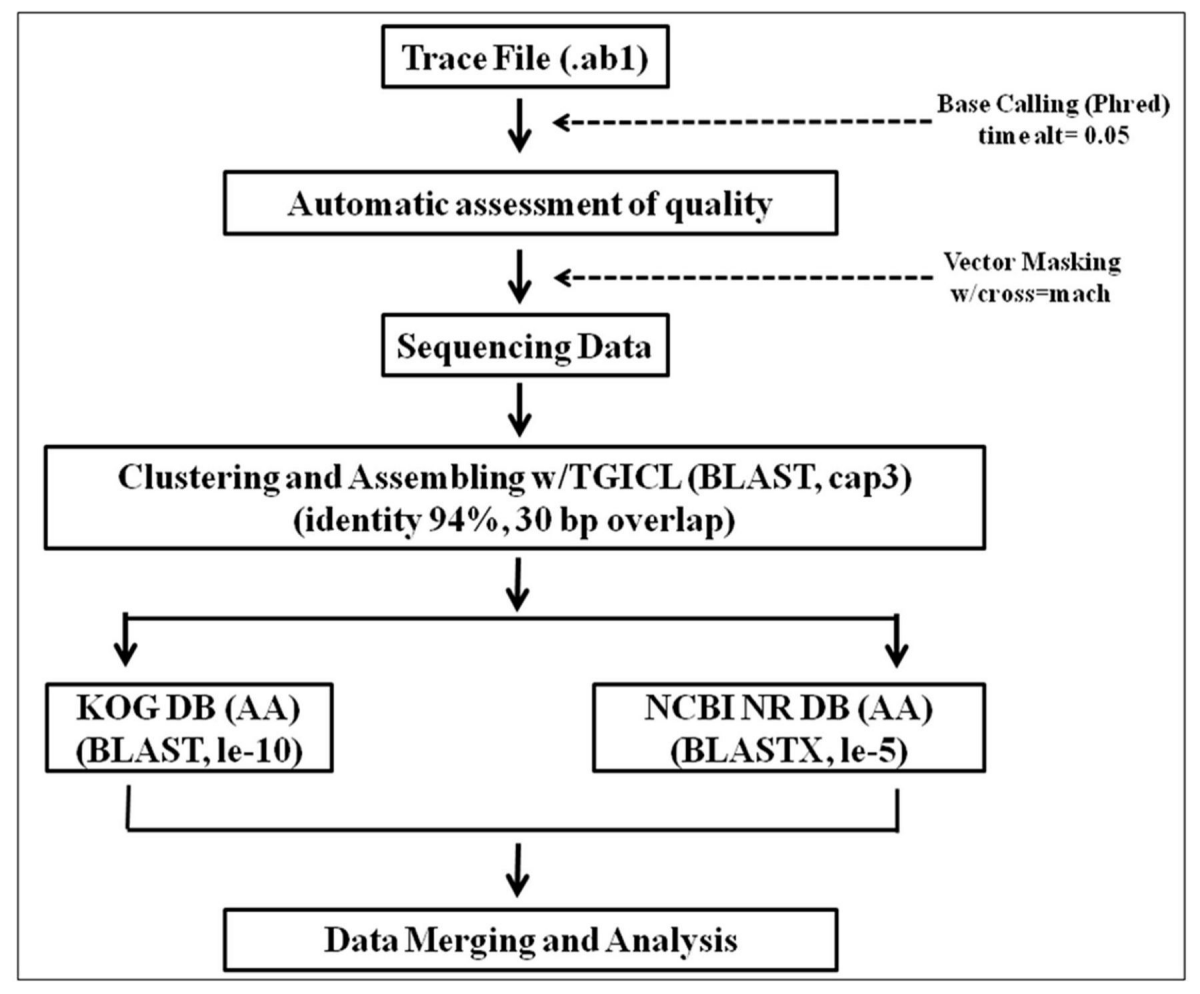
Schematic diagram for EST data analysis.

The Phred software [24,25] was used for base calling and quality assignment of the chromatogram files obtained from the sequencer. The trace files were trimmed using trim-alt 0.05 (Phred score ≥ 20). Cross-match software was used to identify and trim off each vector sequence, and EST sequences shorter than 100 bp were discarded. The TGICL [26] package and cap3 software [27] were used to assemble and cluster ESTs. Finally, the clusters and singletons were analyzed by means of a homology based search using a local BLAST [28] against the NCBI non-redundant (nr) database, NLM, USA. Significant matches were determined when the expected value was <1 x e^-5^ (Figure **1**)**.**

### Functional studies and pathway analysis

The function of ESTs was predicted through KOG (clusters of orthologous groups for eukaryotic complete genomes) analysis [29]. A stand-alone BLAST system (http://www.ncbi.nlm.nih.gov/COG/grace/kognitor.html) was used for functional classification of the reference sequences. Differentially expressed ESTs were categorized into 25 functional groups by the KOG database and the BLASTX program in NCBI (<1 × e^-10^) [29].

DAVID [http://david.abcc.ncifcrf.gov/home.jsp] functional annotation cluster analysis was performed to measure enrichment of the gene ontology (GO) terms within these clusters in MSC, MFC and ALC. Only genes for which at least 5 ESTs were detected were selected from the EST data set, while genes with less than 5 ESTs were excluded from analysis. Additionally, GO terms that reported a p-value of ≤ 0.05 and number of genes ≥ 5 were selected. The GO terms of cellular components, molecular function and biological processes in DAVID were employed to categorize enriched biological categories in three gene lists.

 Pathway mapping for MSC, MFC and ALC was performed using the KEGG Automatic Annotation Server (KAAS) [30]. KAAS offers functional annotation of genes in a genome via a BLAST similarity search against a manually curated set of ortholog groups in the KEGG GENES database. KAAS assigned a KEGG Orthology (KO) number to genes in the data sets, which were mapped to one of KEGG’S reference pathways.

### Identification of MSC, MFC and ALC specific genes

To identify MSC, MFC and ALC specific genes, only genes having at least 5 ESTs in one category and none in the other two categories were selected. For example, MSC specific genes were those with at least 5 ESTs in the MSC category but 0 ESTs in MFC and ALC.

### Real time RT-PCR analysis

Genes with high EST numbers were further studied in C2C12 cells and their expression was confirmed by real time RT-PCR. Briefly, cDNA was synthesized from RNA using Superscript-II reverse transcriptase (Invitrogen). A total of 1μg of RNA (20 µl total volume) was primed with oligo (dT)_20_ primers (Bioneer, Daejeon, Korea), and reverse transcription was then carried out in a thermal cycler by subjecting the samples to 42°C for 50 min and 72°C for 15 min. PCR was subsequently conducted using 2 µl of the 5× diluted cDNA product and 10 pmoles of each gene-specific primer using a 7500 real-time PCR system (Applied Biosystems, CA, USA). Power SYBR® Green PCR Master Mix (Applied Biosystems) was used as the fluorescence source. All primers used were designed with the Primer 3 software (http://frodo.wi.mit.edu) using sequence information listed at the National Center for Biotechnology Information (primer information is provided in Table S1).

### Oil red O staining

Oil red O working solution was prepared asa6:4 dilution of a stock solution (3.5mg/ml Oil red O powder in 100%isopropanol) in distilled water and filtered through Whatman filter paper No. 2. Briefly, 10% formaldehyde-fixed, PBS washed cells were incubated for 1 hr with 1 ml of Oil red O working solution. The cells were then washed with 60% isopropanol and PBS, respectively, after which pictures were taken using a light microscope equipped with a digital camera (Nikon, Tokyo, Japan). To quantify Oil red O in cells, the plates were dried and 500 µl of 100% isopropanol was added to elute the color. The optical density was then measured at 510 nm using a Versa Max microplate reader (Molecular Devices, CA, USA).

### Immunocytochemistry

C2C12 cells in a covered glass-bottom dish were treated with differentiation/transdifferentiation medium and stained for DDAH2 or HBA2 antibody. DDAH2 was stained after 48 hrs and HBA2 was stained after 24 hrs. Bovine MSCs in a covered glass-bottom were cultured at Day 11 and stained with MyoD antibody. Briefly, cells were rinsed with PBS, fixed in 4% formaldehyde, permeabilized by 0.2% TritonX-100,after which the signals were enhanced using an Image-iT^TM^ FX signal enhancer (Invitrogen, CA, USA). The cells were then incubated with rabbit primary antibody (DDAH2/HBA2/MyoD, Santa Cruz Biotechnology, CA, USA) at 4°C in a humid environment overnight. Secondary antibody (Alexa Fluor 488 goat anti-rabbit SFX kit; Molecular Probes, Eugene, OR, USA) was treated for 1 hr at room temperature followed by nuclear stainingwith4´,6´-diamino-2-phenylindole (DAPI; Sigma-Aldrich, MO, USA). Pictures were taken using a fluorescent microscope equipped with a digital camera (Nikon).

### Western blot

Total protein was isolated from cells treated with differentiation or transdifferentiation media and cultured for different lengths of time. Briefly, cells washed with ice-cold PBS and lysed in RIPA lysis buffer with protease inhibitor cocktail (Thermo Scientific, FL, USA) were used for Western blot analysis. Total protein was quantified by the Bradford method using protein assay dye solution [31]. Briefly, 50 µg of protein were electrophoresed in 10% SDS-polyacrylamide gel after reducing at 90°C for 3 min with β-mercaptoethanol and then transferred to a PVDF membrane. Membranes were blocked and hybridized with *DDAH2* (1:500), *HBA2* (1:500), or ß-actin antibody (1:2000) (Santa Cruz Biotechnology, TX, USA) overnight at 4°C. Blots washed in TBST were then incubated with horseradish peroxidase conjugated secondary antibody for an hour at room temperature. Finally, the blots were developed using SuperSignal West Pico Chemiluminescent Substrate (Thermo Scientific, USA).

### Immunohistochemistry

*HBA2* and *DDAH2* expression in bovine tissues was evaluated by immunohistochemistry. Briefly, paraffin-embedded tissue sections were deparaffinized, hydrated, and then quenched for endogenous peroxidase activity in 3% H_2_O_2_ for 15 min. The sections blocked with 5% goat serum were subsequently incubated with either *HBA2* or *DDAH2* antibody (2 μg/mL, Santa Cruz Biotechnology) overnight at 4°C. Next, the sections were washed in PBS three times, after which they were incubated with goat anti-rabbit IgG HRP (Santa Cruz Biotechnology) for 1 hr at RT. Positive signals were visualized by adding diaminobenzidine and hydrogen peroxide as substrates. A negative control experiment was also carried out by omitting the primary antibody. Stained sections were counterstained with hematoxyline, washed in running tap water, and then dehydrated, mounted, and examined using a light microscope. 

### Knockdown of *DDAH2* and HBA2

Transfection of C2C12 cells by siRNA was conducted with either 150 nM of HBA2-siRNA or 50 nM of DDAH2-siRNA (Dharmacon) complexed with 5µl of lipofectamine (Invitrogen) in Optimem (Gibco, NY, USA). Cells were allowed to recover and grow in culture media (DMEM+ 10% FBS+ 1% P/S) until they reached 80% confluence, after which cells were treated in differentiation media (DMEM with 2% FBS and 1% P/S) for *DDAH2* and transdifferentiation media for *HBA2*and grown to the designated time-point.

### Statistical analysis

The normalized mean expression was compared using Tukey’s Studentized Range (HSD) to identify significant differences in gene expression. A nominal *p*-value of less than 0.05 was considered to be statistically significant. Real time RT-PCR data were analyzed by one-way ANOVA using PROC GLM in SAS package ver. 9.0 (SAS Institute, NC, USA).

## Results

### Normalized cDNA libraries and EST analyses

MSCs, MFCs and ALCs cultured from bovine hind leg muscle were used to construct three normalized cDNA libraries. Both the MFCs and ALCs exhibited prominent myotube formation and intracellular lipid accumulation following differentiation and transdifferentiation respectively (Figure S1). During this procedure, we determined the purity of the isolated cells by single cell culture and found that at least 83% were MSCs (data not shown). Nuclear localization of MyoD expression was evident in majority of MSCs when analyzed by immunocytochemistry (Figure S2). Thus, the three normalized cDNA libraries were successfully constructed from MSCs, MFCs, and ALCs. Titration of the libraries resulted in 1.4x10^6^, 5x10^5^, and 3x10^6^ independent clones for MSCs, MFCs, and ALCs, respectively. A total of 24,192 clones (8,064 clones from each library) were randomly selected for DNA sequencing. Vector trimming and elimination of lower quality sequences resulted in a total of 23,919 ESTs that included 7,974 from MSCs, 7,991 from MFCs and, 7,954 from ALCs. Comparison of the success rate of sequencing among these libraries with clusters, singletons, and contigs is shown in Table **1**. The average length (bp) of ESTs was 788, 792, and 776 for MSCs, MFCs, and ALCs, respectively. ESTs assembled and clustered for all the three libraries resulted in 3,333 clusters, 5,517 singletons and 3,842 contigs. 

**Table 1:** Overview of DNA Sequencing.

	**MSC**	**MFC**	**ALC**	**Total**
**Total sequencing trial (Clones)**	8,064	8,064	8,064	24,192
**After editing the sequencing data**	7,974	7,991	7,954	23,919
Total length (bp)	6,381,878	6,451,798	6,371,036	19,204,712
Average length (bp)	788	792	776	
**After sequence clustering and assembly**				
Clusters	1,128	1,139	1,066	3,333
Singletons	2,098	1,750	1,669	5,517
Contigs	1,318	1,287	1,237	3,842

MSC: muscle satellite cell, MFC: myotube-formed cell, ALC: adipocyte-like cell.

### Gene expression in MSCs, MFCs, and ALCs

The ESTs obtained during sequencing were annotated by a BLAST search of the NCBI database. Overall, 20,266 ESTs showed known annotation and 2,526 (940, 686 and 900 for MSC, MFC, and ALC groups, respectively) were unannotated. Gene annotation of the identified ESTs with higher numbers during MSCs, MFCs, and ALCs formation is shown in Table **2**. Among these genes, 37% were also observed by microarray analysis. Genes known to be involved in myogenesis, *myosin regulatory light chain 2* (*MYL2*)*, skeletal muscle isoform* (*MYLPF*) and *actin, alpha skeletal muscle* (*ACTA1*), were only identified during MSC and MFC formation. Similarly, *S100 calcium-binding protein A4* (*S100A4*) and *DDAH2* which have no previous reported role during myogenesis were identified with higher EST numbers during MFC formation. Additionally, *pentraxin related* protein *3* (*PTX3*), *lysophospholipid acyltransferase* (*LPCAT4*) and *HBA2* were identified with high EST numbers during ALC formation. Almost equal expression of fibronectin 1 (FN1) was observed in both MFCs and ALCs.

**Table 2:** List of genes showing more than tenfold difference in ESTs between MSC vs. **MFC/ALC or MFC vs. ALCs**.

Accession ID	MSC	MFC	ALC	M Analysis	Annotation
**NP_001069115.1**	44	33	0	6 (MFC)	Myosin regulatory light chain 2, skeletal muscle isoform (MYLPF)
**AAX37095**	16	0	2	ND	Calmodulin 2 (CaM)
**ABV70623**	14	1	7	2 (ALC)	Cytochrome c oxidase subunit I (Cox1)
**DDA15998.1**	13	1	5	ND	Ribosomal protein L6-like
**NP_001026926.1**	12	9	1	ND	60S ribosomal protein L6 (RPL6)
**NP_776857**	10	1	1	ND	Thioredoxin-dependent peroxide reductase, mitochondrial precursor (AOP-1)
**NP_009207**	10	0	2	ND	Chromobox protein homolog 3 (CBX3)
**NP_001030518.1**	10	6	1	ND	Nucleophosmin (NPM1)
**NP_776493.1**	3	32	13	2 (MFC)	Gap junction alpha-1 protein (GJA1)
**NP_001029607.1**	3	29	6	4 (MFC)	Cathepsin K precursor (CTSK)
**NP_001029876.1**	1	20	0	ND	N(G),N(G)-dimethylargininedimethylaminohydrolase 2 (DDAH2)
**NM_212482.1**	1	28	22	3 (MFC)	Fibronectin1 (FN 1)
**NP_776456.1**	5	17	0	3 (ALC)	Cathepsin B (CTSB)
**DAA25054.1**	0	15	1	ND	Latent-transforming growth factor beta-binding protein 2 (LTBP2)
**ABV70800.1**	4	15	1	ND	NADH dehydrogenase subunit 5 (ND5)
**NP_001014955**	9	14	0	ND	Heat shock protein beta-8 (HSPB8)
**NP_001029607**	1	13	4	ND	Cathepsin K precursor (CTSK)
**NP_776896.1**	1	12	8	2 (MFC)	Metalloproteinase inhibitor 1 precursor (TIMP-1)
**NP_001029505**	0	12	10	2 (MFC)	Diamineacetyltransferase 1 (SAT1)
**NP_777020.1**	2	12	1	2 (MFC)	Protein S100-A4 (S100A4)
**AFH30757**	2	11	0	ND	Tubulin alpha-1B chain (TUBA1B)
**NP_001069392.1**	1	10	9	2 (ALC)	Syndecan-1 precursor (SYND1)
**NP_776331**	3	5	38	ND	Decorin precursor (DCN)
**NP_001019640**	4	10	1	ND	60S ribosomal protein L9 (PGY2)
**NP_776650**	5	10	0	ND	Actin, alpha skeletal muscle (ACTA1)
**NP_004682.2**	3	10	0	ND	V-type proton ATPase subunit d 1 (ATP6V0D1)
**NP_001075044.1**	7	10	0	ND	Desmin (DES)
**NP_00115664**	5	10	0	ND	Selenoprotein M precursor (SELM)
**NP_001069727.1**	0	0	88	3 (MFC)	Pentraxin-related protein PTX3 precursor (PTX3)
**NP_776502.1**	0	4	41	9 (MFC)	Glutathione peroxidase 3 precursor (GPX3)
**DAA32273**	1	0	31	17 (MFC)	Plasminogen activator inhibitor type 1, member 2 (SERPINE2)
**NP_786976.1**	14	2	24	2 (MFC)	Galectin-1 (Gal-1)
**XP_519048**	0	10	19	ND	Secreted frizzled-related protein 4 isoform 2 (SFRP4)
**ABM06155.1**	2	0	17	11 (MFC)	Adipose differentiation-related protein (PLIN2)
**NP_001070890.2**	0	0	15	5 (MFC)	Hemoglobin subunit alpha (HBA)
**NP_776327**	0	3	14	ND	Clusterinpreproprotein (CLU)
**NP_001071369.1**	0	0	13	ND	Lysophospholipidacyltransferase (LPCAT4)
**AAC95151**	0	20	26	ND	Serine protease HTRA1 (HTRA1)
**NP_001028934.1**	3	1	12	ND	Rab GDP dissociation inhibitor beta (GDI2)
**CAI24449**	1	2	12	ND	Member RAS oncogene family (RAB1)
**NP_776739**	0	0	12	11 (MFC)	Fatty acid-binding protein 4 (FABP4)
**NP_001029956**	0	0	11	ND	Mitochondrial fission 1 protein (FIS1)
**NP_000971.1**	1	4	10	ND	60S ribosomal protein L18a (RPL18A)

Numbers indicate ESTs. M analysis represents fold differences in mRNA expression of genes and ND are genes not detected by DNA microarray. MFC and ALC represent fold difference of myotube-formed cells and adipose-like cell during microarray analysis, respectively.

### Functional study and pathway analysis of ESTs

**Figure 2:**
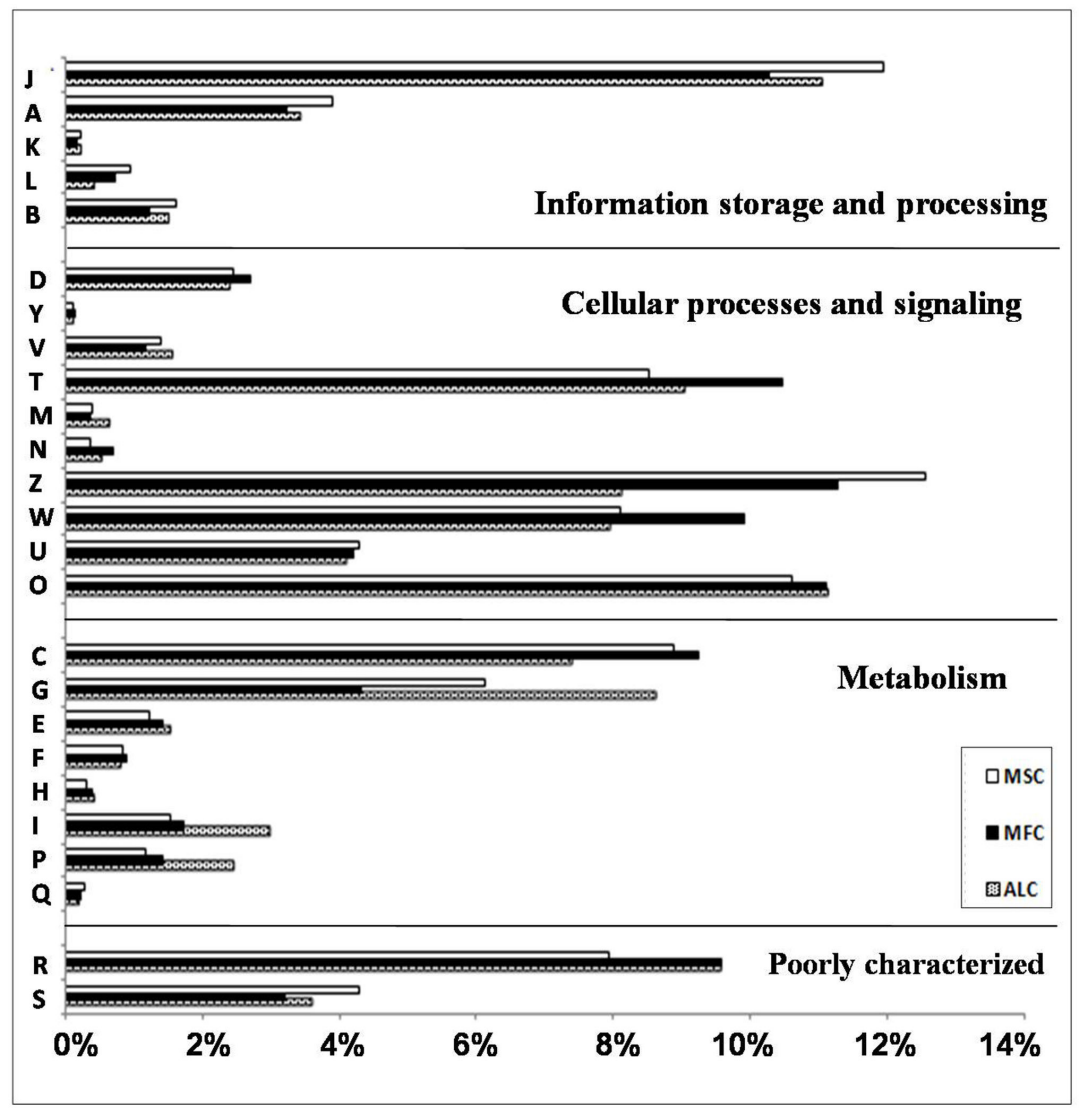
KOG analysis of ESTs in MSC, MFC and ALC. The functions of genes were categorized and each function is represented by the symbols given below: . [J] Translation, ribosomal structure and biogenesis, [A] RNA processing and modification, [L] replication, recombination and repair, [B] chromatin structure and dynamics, [Y] nuclear structure, [V] defense mechanisms, [T] signal transduction mechanisms, [M] cell wall/membrane/envelope biogenesis, [N] cell motility, [Z] cytoskeleton, [W] extracellular structures, [U] intracellular trafficking, secretion, and vesicular transport, [O] posttranslational modification, protein turnover, chaperones, [C] energy production and conversion, [G] carbohydrate transport and metabolism, [E] amino acid transport and metabolism, [F] nucleotide transport and metabolism, [H] coenzyme transport and metabolism, [I] lipid transport and metabolism, [P] inorganic ion transport and metabolism, [R] general function prediction only, [S] function unknown. The X-axis represents percentage and Y axis represents functional category.

The differentially expressed genes identified by ESTs were functionally categorized by querying the NCBI eukaryotic Orthologous Group (KOG) database. Twenty-five different functional classes were formulated and summarized into four functional groups, information storage and processing, cellular processes and signaling, metabolism, and poorly characterized. A total of 16,048 ESTs (MSCs=5,534, MFCs=5,265, ALCs=5,249) were analyzed using the KOG database, among which the highest percentage was related to cellular processes and signaling. Moreover, genes related to translation, ribosomal structure and biogenesis, posttranslational modification, protein turnover, chaperones and energy production and conversion were enriched during MSCs, MFCs and ALCs formation (Figure **2**). A large number of ESTs represented genes related to signal transduction, cytoskeletons and extracellular structures (Table **3A**) during MFC formation. *Latent transforming growth factor beta binding protein 2* (*LTBP2*), *tubulin alpha-1B chain* (*TUBA1B*) and *40S ribosomal protein SA* (*RPSA*) were found to be the genes with the highest ESTs in these categories. Similarly, during ALC formation, ESTs related to lipidtransport and metabolism, carbohydrate transport and metabolism and energy production and metabolism were abundant (Table **3B**). *Fatty acid binding* protein *4* (*FABP4*), *2-oxoglutarate dehydrogenase* and *HBA2* showed the highest EST numbers. In contrast, *transgelin* (*TAGLN*)*,* osteonectin (ON) and *cytoskeletal beta actin* showed almost equal numbers of ESTs in MSCs, MFCs and ALCs (data not shown), suggesting their equal contribution during MSC differentiation and transdifferentiation.

**Table 3:** Functional classifications of genes with more than 3 fold differences in ESTs.

	**Acession ID**	**KOG**	**MSC**	**MFC**	**ALC**	**Annotation**
**A) MFC**	DAA25054.1	Signal transduction mechanisms	0	15	1	Latent transforming growth factor beta binding protein 2 (LTBP2)
	NP_001094594.1		0	8	2	Death-associated protein kinase 3 (DAPK3)
	AAP44493.1		0	6	0	Aggrecan (ACAN)
	NP_001029224.1		1	6	1	Growth hormone inducible transmembrane protein (GHITM)
	NP_001092564.1		0	6	0	Ras-related protein Rab-7L1(GHITM)
	NP_001033199.1		1	5	1	Vasodilator-stimulated phosphoprotein (VASP)
	NP_001069325.1		0	5	0	Serine/threonine-protein phosphatase 2A catalytic subunit beta isoform (PPP2CB)
	NP_001076899.1		0	5	0	LETM1 domain-containing protein 1 (LETMD1)
	NP_001095955.1		1	5	0	Rho GTPase activating protein 29 (ARHGAP29)
	NP_776686.1		0	5	0	Calpain, small subunit 1 (CAPNS1)
	AFH30757	Cytoskeleton	2	11	0	Tubulin alpha-1B chain (TUBA1B)
	NP_080645		0	7	0	Actin-related protein 2/3 complex subunit 5 (ARPC5)
	ABM06144		0	5	0	Myosin regulatory light polypeptide 9 (MYL9)
	NP_001069115		0	5	0	Myosin regulatory light chain 2, skeletal muscle isoform (MYLPF)
	XP_003586919		0	4	0	Predicted: plectin-like Protein (PITG)
	NP_001029885		1	4	1	Actin-related protein 2/3 complex subunit 2 (ARPC2)
	DAA16986		0	4	0	Filamin B, beta isoform 3 (FLNB)
	NP_001098733.1	Extracellular structures	4	14	4	40S ribosomal protein SA (RPSA)
	NP_001178350.1		1	5	1	Receptor-associated protein of the synapse (RAPSN)
	XP_854073.1		0	3	0	Complement C1q tumor necrosis factor-related protein 3 isoform 1 (C1QTNF3)
**B) ALC**	NP_001071369.1	Lipid transport and metabolism	0	0	13	Lysophospholipidacyltransferase (LPCAT4)
	NP_776739.1		0	0	12	Fatty acid binding protein 4 (FABP4)
	NP_001071580.1		0	0	7	Acyl-CoA synthetase family member 2, mitochondrial precursor (ACSF2)
	NP_001039878.1		0	0	5	3-hydroxyisobutyryl-Coenzyme A hydrolase (HIBCH)
	AAL99940.1		0	0	4	Stearoyl-CoA desaturase (SCD)
	NP_001073761.1		0	0	4	Short-chain dehydrogenase/reductase family 42E member 1 (SDR42E1)
	NP_001073689		0	0	4	Hormone-sensitive lipase (LIPE)
	NP_001069498.1	Carbohydrate transport and metabolism	2	0	7	2-oxoglutarate dehydrogenase, mitochondrial precursor (OGDH)
	NP_001095385		0	0	4	Fructose-bisphosphatealdolase A (ALDOA)
	NP_001231064.1		0	0	4	Glucose-6-phosphate dehydrogenase (G6PD)
	AAC16069.1		0	1	6	Glyceraldehyde-3-phosphate dehydrogenase (GAPC1)
	NP_001070890	Energy production and metabolism	0	0	15	Hemoglobin subunit alpha (HBA)
	NP_001017954		2	2	8	V-type proton ATPase 16 kDaproteolipid subunit (ATP6V0C)
	NP_001039791		0	0	4	Mitochondrial ornithine transporter 1 (SLC25A15)
	NP_001002891.1		0	1	4	Cytochrome c oxidase subunit 5A, mitochondrial precursor (COX5A)
	NP_001069834.1		0	0	4	BCL2/adenovirus E1B 19 kDa protein-interacting protein 3 (BNIP3)
	AAI04512		0	0	3	ATP6AP1 protein (ATP6AP1)
	NP_001033671		0	0	3	Electron transfer flavoprotein subunit beta (ETFB)

A) MFC vs. MSC/ALC and B) ALC vs. MSC/MFC by KOG. Numbers indicate ESTs.

To classify biological processes coordinately regulated during MSC, MFC and ALC formation, we further created three subsets of all highly expressed genes by keeping those genes for which the number of ESTs was ≥ 5. All other genes with < 5 ESTs were discarded. These highly expressed subsets consisted of 233 MSC (MSC233), 258MFC (MFC258), and 248ALC (ALC248) genes, respectively. These three highly expressed gene lists were then used for functional annotation by employing the Functional Annotation Cluster (FAC) tool available in the Database for Annotation, Visualization, and Integrated Discovery (DAVID) [http://david.abcc.ncifcrf.gov/home.jsp]. Table S2 provides the list of these highly expressed subsets and the genes annotated by DAVID. The GO terms “Biological Process,” “Cellular Component” and “Molecular Function” were used for annotations. DAVID FAC analysis of MFC258 produced a total of 41 functional clusters using default parameters. Similarly, FAC analysis of MSC233 genes resulted in 22 clusters, whereas 31 clusters were reported for ALC248 genes. GO terms having at least ten genes from the resulting functional analysis with statistically significant p-values for these three subsets are listed in Table **4** and a complete list is available in Table S3. Among these categories, genes involved in the extracellular region, extracellular matrix (ECM), structural molecule activity, non-membrane-bounded organelle, cytoskeleton, calcium ion binding, ribonucleoprotein complex and various carbohydrate metabolic processes were the most represented groups, indicating that the cells were undergoing rapid structural rearrangement for cellular differentiation. High enrichment of GO terms such as ECM, cytoskeleton, and carbohydrate metabolism were found in all the three sets. However, some specific terms for each category were also identified. MFC showed a firm preference for terms that involve adhesion, endopeptidase activity and embryonic development. Similarly, MSC includes processes such as cellular homeostasis, oxidative phosphorylation, as well as myofibril and contractile fiber, whereas ALC had a strong inclination toward phosphate metabolic processes and kinase activity. Commencement of differentiation in cells leads to intense alterations in the transduction of locomotion and cell shape controlling proteins and depends on reorganization of their cytoskeleton and plasma membranes [32]. 

**Table 4:** DAVID Functional Annotation Cluster Analysis.

	**GO Term**	**No. of Genes**	**p-value**
**A) MSC**	GO:0043232~intracellular non-membrane-bounded organelle	23	0.0062
	GO:0043228~non-membrane-bounded organelle	23	0.0062
	GO:0005198~structural molecule activity	21	0.0000
	GO:0005576~extracellular region	16	0.0676
	GO:0044421~extracellular region part	14	0.0008
	GO:0006091~generation of precursor metabolites and energy	13	0.0000
	GO:0030529~ribonucleoprotein complex	13	0.0004
	GO:0006412~translation	12	0.0002
	GO:0031012~extracellular matrix	12	0.0000
	GO:0005840~ribosome	11	0.0001
	GO:0005578~proteinaceous extracellular matrix	11	0.0000
	GO:0003735~structural constituent of ribosome	10	0.0001
**B) MFC**	GO:0005576~extracellular region	28	0.0001
	GO:0043232~intracellular non-membrane-bounded organelle	28	0.0058
	GO:0043228~non-membrane-bounded organelle	28	0.0058
	GO:0044421~extracellular region part	25	0.0000
	GO:0031012~extracellular matrix	21	0.0000
	GO:0005578~proteinaceous extracellular matrix	20	0.0000
	GO:0005198~structural molecule activity	20	0.0000
	GO:0005509~calcium ion binding	20	0.0000
	GO:0005856~cytoskeleton	17	0.0081
	GO:0044420~extracellular matrix part	13	0.0000
	GO:0008092~cytoskeletal protein binding	13	0.0000
	GO:0022610~biological adhesion	11	0.0030
	GO:0007155~cell adhesion	11	0.0030
	GO:0006412~translation	10	0.0059
	GO:0030529~ribonucleoprotein complex	10	0.0646
	GO:0001568~blood vessel development	10	0.0000
	GO:0001944~vasculature development	10	0.0000
	GO:0004175~endopeptidase activity	10	0.0140
**C) ALC**	GO:0005576~extracellular region	23	0.0008
	GO:0044421~extracellular region part	20	0.0000
	GO:0031012~extracellular matrix	17	0.0000
	GO:0005198~structural molecule activity	17	0.0000
	GO:0005578~proteinaceous extracellular matrix	16	0.0000
	GO:0005509~calcium ion binding	12	0.0306
	GO:0006091~generation of precursor metabolites and energy	11	0.0001
	GO:0030529~ribonucleoprotein complex	11	0.0094

A) MSC233, B) MFC258 and C) ALC248. GO terms having at least 10 genes from the resulting functional clusters and statistically significant p-values are shown.

**Figure 3:**
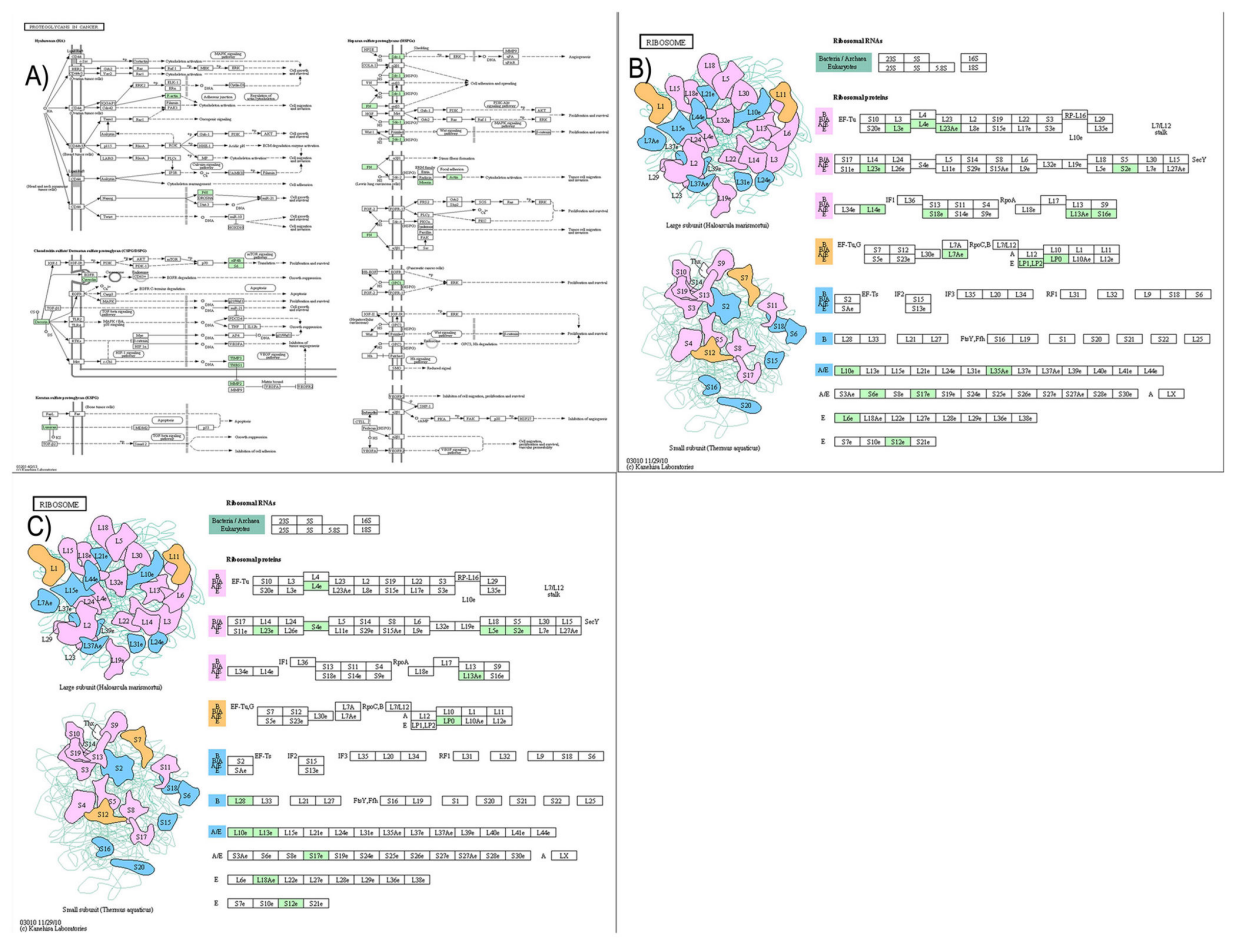
Biochemical pathway analysis of highly expressed gene list. Representative KEGG pathway for A) MSC, B) MFC and C) ALC.

We also identified the biochemical pathways of MFC258, MSC233 and ALC248 genes annotated in the present study. FASTA formatted amino acid sequences of DAVID annotated genes in these sets were fed into the KAAS for prediction of various pathways. A total of 130 pathways were predicted for MFC258, whereas 114 and 128 pathways were predicted for MSC233 and ALC248, respectively. A representative pathway for each of the three categories is shown in Figure **3** and a complete list of all pathways is provided in Table S4. Proteins involved in various stages of cellular differentiation pathways including proteoglycans in cancer, regulation of actin cytoskeleton, focal adhesion, tight junction, ribosome, oxidative phosphorylation, ECM-receptor interaction, and various important signaling pathways including the MAPK signaling, Wnt signaling, Hippo signaling, TGF-beta signaling, PI3K-Akt signaling and calcium signaling pathways were represented by unigenes derived from our EST dataset. These data provide substantial evidence that the ESTs generated in this study offer an important resource for MSC differentiation related gene discovery and future functional studies.

### Identification of MSC, MFC and ALC specific genes

We also attempted to determine if our EST dataset represented MSC, MFC and ALC specific genes (e.g., genes that were detected in MFC only and absent from MSC and ALC). Based on the criteria mentioned in the methods section, we identified 25, 28 and 36 genes that were present in MSC, MFC and ALC only (Table **5**). From this table, extremely high expression of *PTX3*, *HBA* and *LPCAT4* was observed in ALC only. Similarly, *adapter-related protein* complex *3 mu-1 subunit* (*AP3M1*) and *60S ribosomal protein L3* (*RPL3*) were among a few genes expressed in MFC and MSC, respectively. Over-representation of these MSC, MFC and ALC specific genes (esp. *PTX3* and *HBA*) reflects their distinctiveness and indicates that they may serve as potential candidates for future studies conducted to elucidate their role in muscle cell differentiation. 

**Table 5:** List of genes specific to A) MSC, B) MFC and C) ALCs.

	**ID**	**MSC**	**MFC**	**ALC**	**Description**
**A) MSC**	NP_777140.1	8	0	0	60S ribosomal protein L3
	BAE35030.1	7	0	0	Leucine-rich repeat-containing protein 59
	NP_001040078.1	7	0	0	Cleft lip and palate transmembrane protein 1 homolog
	NP_001095606.1	7	0	0	Nucleoredoxin
	XP_613708.3	7	0	0	Cartilage-associated protein
	CAH56277.1	6	0	0	Hypothetical protein
	EDL84232.1	6	0	0	Tropomyosin alpha-1 chain
	NP_001093789.1	6	0	0	Ubiquitin carboxyl-terminal hydrolase 4
	NP_001095592.1	6	0	0	Translocon-associated protein subunit alpha
	XP_002118239.1	6	0	0	Predicted protein
	XP_540101.2	6	0	0	Rho-related GTP-binding protein RhoB
	XP_874996.2	6	0	0	Oxysterol-binding protein-related protein 5
	AAI02075.1	5	0	0	60S acidic ribosomal protein P0
	ACE75861.1	5	0	0	Troponin I, slow skeletal muscle
	BAG57729.1	5	0	0	WD repeat and SOCS box-containing protein 1
	EAW68950.1	5	0	0	ELAV-like protein 1
	EAW83679.1	5	0	0	Calumenin
	EAW91832.1	5	0	0	Zinc finger protein 706
	NP_001039509.1	5	0	0	UMP-CMP kinase
	NP_001094577.1	5	0	0	FARSA protein
	NP_777109.1	5	0	0	ATP synthase subunit alpha, mitochondrial
	NP_861528.1	5	0	0	tRNA (cytosine-5-)-methyltransferase
	XP_001082579.1	5	0	0	Cofilin-2
	XP_001380719.1	5	0	0	NA*
	XP_001788533.1	5	0	0	Integrin alpha-7
	XP_001790312.1	5	0	0	Zinc finger MYM-type protein 1
**B) MFC**	BAG61348.1	0	8	0	Adapter-related protein complex 3 mu-1 subunit
	NP_001069211.1	0	7	0	Translation initiation factor eIF-2B subunit alpha
	NP_001106692.1	0	7	0	RTN4 protein
	Q9CPW4.3	0	7	0	Actin-related protein 2/3 complex subunit 5
	XP_001495008.1	0	7	0	transcription from RNA polymerase II promoter
	XP_541506.2	0	7	0	Nucleobindin 1 precursor isoform 1
	AAP44493.1	0	6	0	Aggrecan
	NP_001029707.1	0	6	0	NAD(P)H dehydrogenase, quinone 1
	NP_001032534.1	0	6	0	ATP-citrate synthase
	NP_001092564.1	0	6	0	RAB7L1 protein
	XP_001255518.2	0	6	0	PREDICTED: zinc finger protein 106 homolog
	XP_871977.3	0	6	0	Uncharacterized protein
	XP_875886.2	0	6	0	PREDICTED: protein FAM101B, partial
	AAI12879.1	0	5	0	Dehydrogenase/reductase SDR family member 4
	ABM06144.1	0	5	0	Myosin regulatory light polypeptide 9
	BAG56803.1	0	5	0	RWD domain-containing protein 4 (FAM28A)
	EAW78788.1	0	5	0	hCG1806964, isoform CRA_b
	NP_001029875.1	0	5	0	Elongation of very long chain fatty acids
	NP_001069325.1	0	5	0	Serine/threonine-protein phosphatase 2A catalytic subunit beta isoform
	NP_001076899.1	0	5	0	LETM1 domain-containing protein 1
	NP_001096653.1	0	5	0	Polymerase (DNA-directed), delta interacting protein 3
	NP_776686.1	0	5	0	Calpain small subunit 1
	NP_874363.1	0	5	0	Selenoprotein V
	P12624.6	0	5	0	Myristoylated alanine-rich C-kinase substrate
	P13605.2	0	5	0	Fibromodulin
	XP_001371068.1	0	5	0	Myosin regulatory light chain 2
	XP_001491993.2	0	5	0	PREDICTED: LOW QUALITY PROTEIN: alpha-actinin-2
	XP_875656.2	0	5	0	zinc finger MYM-type protein 6
**C) ALC**	NP_001069727.1	0	0	88	Pentraxin-related protein PTX3
	NP_001070890.2	0	0	15	Hemoglobin subunit alpha
	NP_001071369.1	0	0	13	LPCAT4 protein
	NP_776739.1	0	0	12	Fatty acid-binding protein, adipocyte
	XP_001143081.1	0	0	11	Mitochondrial fission 1 protein
	EDL24540.1	0	0	9	Prostaglandin E synthase 3
	NP_001039457.1	0	0	9	Neugrin
	1Z2W	0	0	8	Vacuolar protein sorting-associated protein 29
	ACD50133.1	0	0	8	Interleukin-1 receptor-associated kinase 2 transcript variant 1
	NP_001096812.1	0	0	8	TSC22D3 protein
	AAB35870.1	0	0	7	Dual specificity protein phosphatase
	BAD92273.1	0	0	7	Proteasome 26S ATPase subunit 5 variant
	NP_001033273.1	0	0	7	Retrograde Golgi transport protein RGP1 homolog
	NP_001033592.1	0	0	7	Translocon-associated protein subunit delta
	NP_001039816.1	0	0	7	Thyroid hormone receptor interactor 4
	NP_001071580.1	0	0	7	Acyl-CoA synthetase family member 2, mitochondrial
	XP_001366185.1	0	0	7	Metastasis-associated protein MTA2
	XP_001367952.1	0	0	7	Heterogeneous nuclear ribonucleoprotein A0
	XP_001790184.1	0	0	7	Ubiquitin carboxyl-terminal hydrolase 30
	NP_001029777.1	0	0	6	KxDL motif-containing protein 1
	AAV97884.1	0	0	5	Mitogen-activated protein kinase kinase kinase kinase 4 isoform
	BAG53140.1	0	0	5	Mortality factor 4-like protein 1
	CAA28542.1	0	0	5	Clathrin light chain A
	EDM09101.1	0	0	5	eukaryotic translation initiation factor 4E binding protein 1
	NP_001001598.1	0	0	5	Prolyl 4-hydroxylase subunit alpha-3
	NP_001030280.1	0	0	5	Cytosolic non-specific dipeptidase
	NP_001039878.1	0	0	5	3-hydroxyisobutyryl-CoA hydrolase, mitochondrial
	NP_001068735.1	0	0	5	Canopy 2 homolog (Zebrafish)
	NP_001068999.1	0	0	5	Thiopurine S-methyltransferase
	NP_001076071.1	0	0	5	DDX41 protein
	XP_001102980.1	0	0	5	Spermatogenesis-defective protein 39 homolog
	XP_001170347.1	0	0	5	NA*
	XP_001251315.2	0	0	5	Small integral membrane protein 3
	XP_001492130.2	0	0	5	Reticulocalbin-3
	XP_611630.4	0	0	5	Collagen alpha-1(XII) chain
	XP_849800.1	0	0	5	NA*
	ZP_01925762.1	0	0	5	NA*

NA*: This record was removed from NCBI as a result of standard genome annotation processing.

### mRNA and protein expression

**Figure 4:**
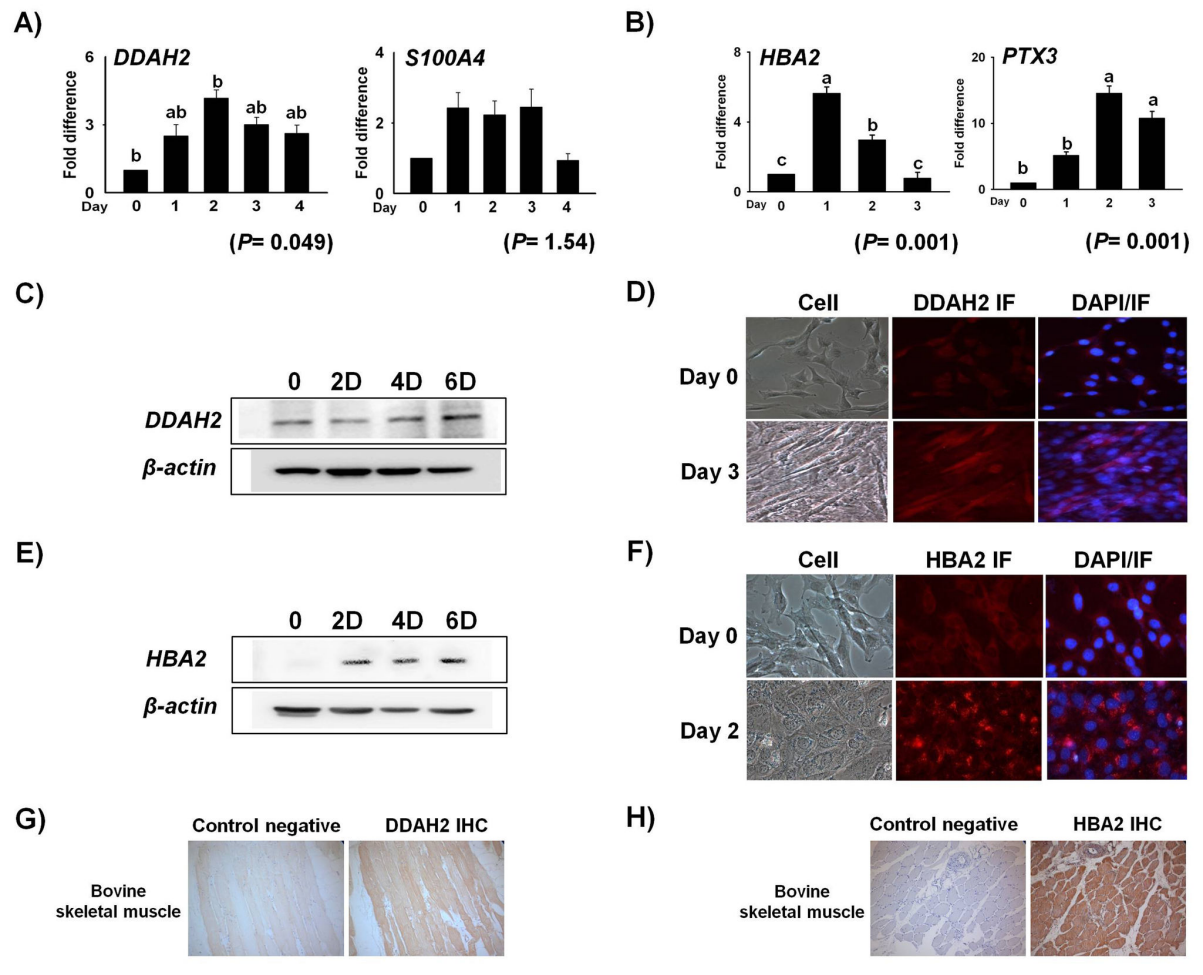
mRNA expression of genes identified with higher EST numbers. C2C12 cells treated with differentiation and transdifferentiation media at 80% confluence were harvested at different time points. Real time PCR was carried out with cDNA synthesized from1µg of total RNA. mRNA expression analysis of most of the genes showed more than 2 fold induction during MFCs (A) and ALCs (B) formation, respectively. Western blot analysis of *DDAH2* shows a gradual increase in protein expression with time (C). Cellular localization of DDAH2 by immunocytochemistry during myogenesis. The first column shows cell pictures at Day 0 and Day 3. The second column shows expression of *DDAH2* and the third column shows a merged image of DAPI-stained nuclei and *DDAH2* IF (D).Western blot analysis of *HBA2* expression during transdifferentiation confirms its protein level expression (E). Cytoplasmic localization of HBA2 by immunocytochemistry during transdifferentiation. The first column shows cell pictures at Day 0 and Day 2, the second column shows expression of *HBA2* IF and the third column shows a merged image of DAPI-stained nuclei and *HBA2* IF (F). DDAH2 and HBA2 immunohistochemistry of bovine skeletal muscle (G and H). Day 0 represents control (mean ± S.D., n= 3). *p*-value indicates the statistical significance of the data and different letters show significant differences among groups.

Expression of identified genes that showed high EST numbers was validated by real time RT-PCR. mRNA expression was checked at different time points during differentiation and transdifferentiation of C2C12 cells. *DDAH2* showed more than 4 fold induction at Day 2 when compared to Day 0, while *S100A4* maintained its pattern of expression from Day 1 to 3 during MFC formation (Figure **4A**). *PTX3* mRNA expression reached as high as 12 fold on Day 2, whereas *HBA2* reached almost 5 fold at Day 1during ALC formation (Figure **4B**). Protein expression of the two representative genes was confirmed by immunoblot and its cellular localization was visualized by immunocytochemistry in C2C12 cells. A time dependent increase in the concentration of protein was observed by Western blot (Figure **4C**) and increased cytoplasmic expression of DDAH2 protein was evident upon immunocytochemistry (Figure **4D**). Similarly, expression of HBA2 was detected on transdifferentiated C2C12 cells from as early as day 1, and gradually increased to day 3 (Figure **4E**). Moreover, clear localization of cytoplasmic HBA2 protein was evident at day 2 upon immunocytochemistry analysis (Figure **4F**). Immunohistochemistry of DDAH2 and HBA2 on tissue sections from bovine skeletal muscle revealed *in vivo* muscle expression of these proteins (Figure **4G and H**).

### Knockdown of *DDAH2* and *HBA2*

**Figure 5:**
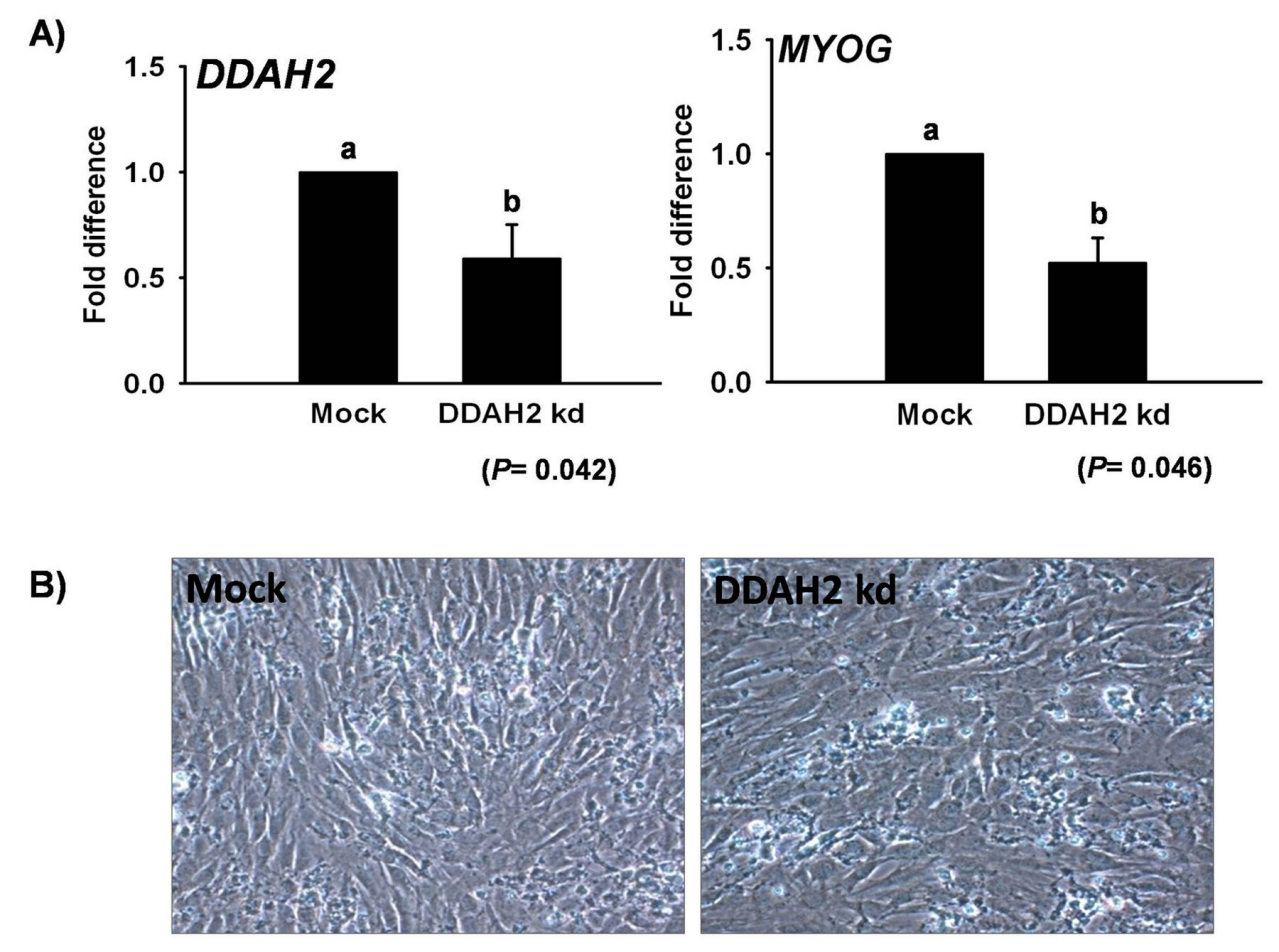
*DDAH2* knockdown in C2C12 cells during myogenesis. mRNA expression of *DDAH2* and *MYOG* after *DDAH2*_kd_ during differentiation in C2C12 at Day 2 (A). Representative cell picture showing morphological changes in *DDAH2*_kd_ cells (B). Mock represents control (mean ± S.D., n= 3). *p*-value indicates the statistical significance of the data and different letters show significant differences among groups.

**Figure 6:**
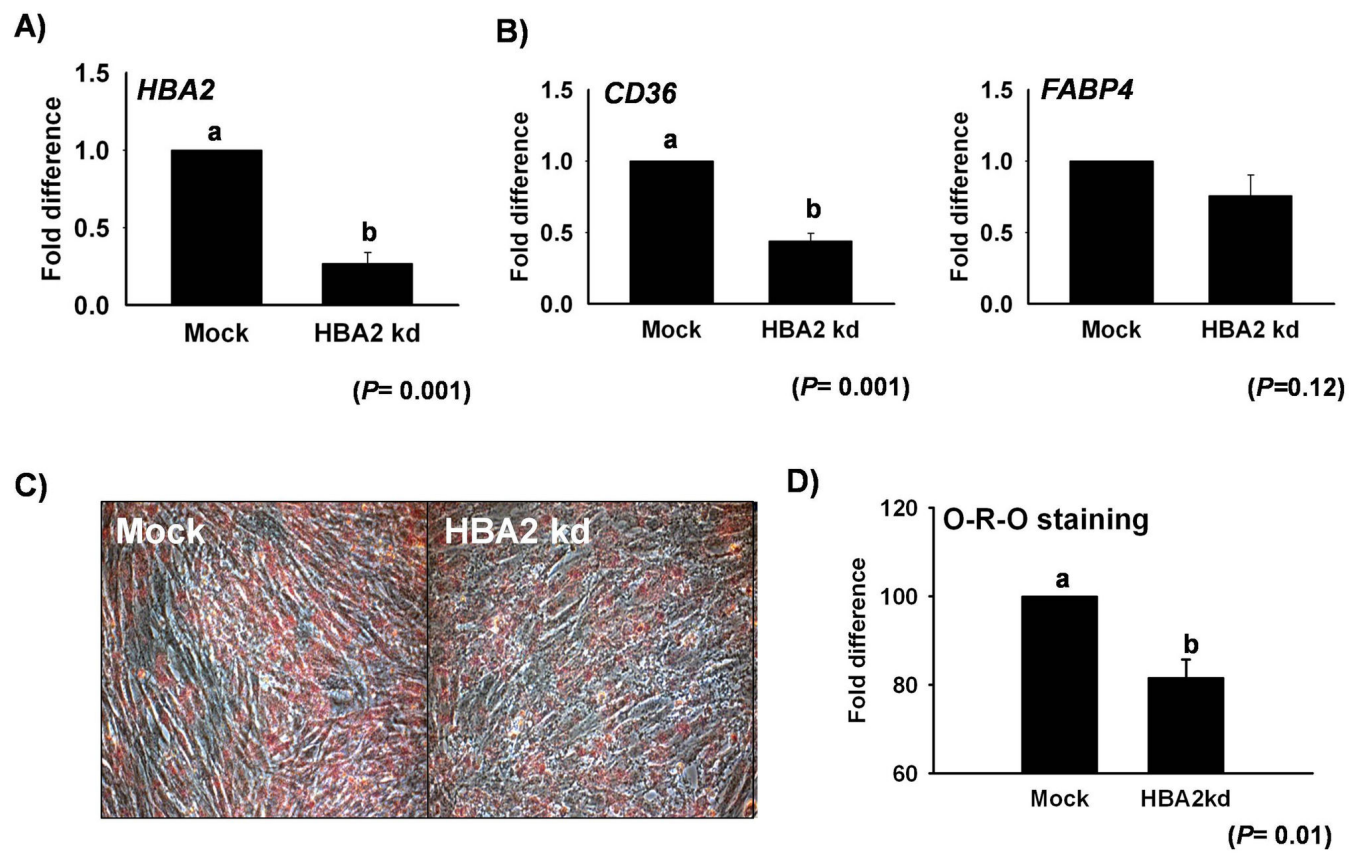
*HBA2* knockdown in C2C12 cells during transdifferentiation. (A) mRNA expression of *HBA2* after *HBA2*_kd_ during transdifferentiation in C2C12 at Day 1, and (B) *HBA2*_kd_ effect on mRNA expression of different adipogenic marker genes.(C) Cell picture following intracellular lipid staining by O-R-O on Day 5 during transdifferentiation in HBA2_kd_ and Mock cells. Quantification of O-R-O at 510 nm. Control represents Mock (mean ± S.D., n= 3). *p*-value indicates the statistical significance of the data and different letters show significant differences among groups.

To confirm the role of genes identified during differentiation and transdifferentiation, gene knockdown by siRNA was employed in *DDAH2*and *HBA2*. DDAH2_kd_ by siRNA showed a 40% reduction in mRNA expression with a slight change in cell morphology (Figure **5A&B**). Interestingly, DDAH2_kd_ showed a decrease in *MYOG* upto 50% during myogenesis (Figure **5A**). Furthermore, *HBA2* silencing in C2C12 cells showed an upto 70% reduction in its expression during transdifferentiation (Figure **6A**). *CD36*, which is a marker gene for adipogenesis, was checked in HBA2_kd_ and its expression was found to be reduced up to 50%, whereas *FABP4* was slightly decreased (Figure **6 B**). The effects of HBA2_kd_ on intracellular lipid formation followed by its treatment with transdifferentiation media were also evaluated at day 5 by Oil Red O (O-R-O) staining and the results showed a 20% reduction in intracellular fat during ALC formation (Figure **6C& D**).

## Discussion

Cultures of bovine MSCs in media containing fetal bovine serum obtained from the same species may have an added advantage because they can mimic the *in vivo* environment more closely [21]. Identification of many genes differentially expressed during MFCs formation and ALCs formation using microarray analysis in association with the primary bovine MSCs culture system and validation of the physiological role of each gene support this idea [6,20,33]. Herein, we describe normalized cDNA libraries constructed to newly identify additional genes that were not identified in our previous microarray analysis and to maximize the number of unique EST sequences from bovine MSCs, MFCs, and ALCs. 

*MYLPF*, a myogenic marker gene [34], *connexin 43* (*CX43*), a gap junction protein [35], and *desmin*, a cycloskeletal muscle-specific gene [36], were identified during MFC formation, confirming the reliability of our library. *DDAH2* expressed in endothelial cells, which has been found to reduce endothelial nitric oxide (eNO) generation [37], *Selenoprotein M precursor* (*SELM*), which is known to cause apoptotic cell death [38], and *S100A4*, which is involved in regulation of cell cycle progression [39], were reported during myogenesis for the first time in this study. Furthermore, identification of genes known to be expressed during adipogenesis including *FABP4* [40] and stearoyl-CoA desaturase (SCD) [41] during ALC formation also confirmed our results. *PTX3*, which is responsible for inflammation [42], and *LPCAT4*, which catalyzes membrane phospholipids [43] were also identified. Identification of *LPCAT4* in skeletal muscle lipid droplet protein [44] indicates that it plays a putative role in ALC formation. Moreover, expression of *serine protease HTRA1*, FN *1*, and *diamine acetyltransferase1* is common during MFC and ALC formation, suggesting the existence of a common pathway between these two processes. Similarly, the involvement of *PRDM16* in a bidirectional cell fate switch between skeletal myoblasts and brown adipocytes was previously reported [45]. KOG analysis conducted to assign biological functions indicated a total of 16,048 and 3,680 sequences with known and unknown functions, respectively. Further studies of annotated genes may enable identification of novel genes involved in MFC and ALC formation. However, KOG analysis clearly indicated that most ESTs were related to genes responsible for cellular and signaling processes and cytoskeleton in all MSCs, MFCs and ALCs. To further authenticate the enrichment of processes involved in differentiation, DAVID functional analysis was performed on three highly expressed EST subsets, MSC233, MFC258 and ALC248. Myogenesis is a process in which muscle satellite cells are required to activate, proliferate, and differentiate to form multinucleated myofibers. These processes involve cell adhesion, migration, and cell to cell interactions that are altered by positive and negative signals from the extrinsic extracellular environment [46]. High enrichment of ECM GO terms is evident from the fact that communication among muscle cells and ECM plays a fundamental role in the regulation of proliferation and differentiation processes. Proteoglycans represent an essential group of ECM molecules that are vital in signal transduction and supporting the structure and function of a tissue [46]. Several of these ECM proteoglycans in our EST dataset such as *glypican-1* and *decorin* showed high expression rates. *Glypican-1* plays an essential role in the growth and development of muscle by regulating fibroblast growth factor 2 (FGF2) [47]. Myoblasts without any *glypican-1* expression show imperfect differentiation and decreased expression of *myogenin*, *myosin* and myoblast fusion index [47,48]. Similarly, *decorin* interacts with *TGF-β1*, a strong inhibitor of myoblast proliferation and differentiation [49], to modulate TGF-β1-dependent cell growth stimulation or inhibition [50]. 

It is well known that extensive cytoskeleton rearrangement occurs during myoblast differentiation into multinucleated muscle fiber [51]. High expression of genes such as *Cdc42 effector* protein *3* (*CDC42EP3*) and *myristoylated alanine-rich C-*kinase *substrate* (*MARKS*), which are involved in actin cytoskeleton organization [52] and regulation [53], was verified by our data, which showed higher ESTs during MFC formation.Carbohydrate metabolism also plays a significant role throughout myogenic differentiation [54], and the greater abundance of enzymes such as phosphorylase, glyceraldehyde-3-phosphate dehydrogenase, phosphoglycerate kinase 1 and alpha-enolase involved in carbohydrate metabolism observed in our study supports their role in differentiation. In addition to some common biological processes in MSC, MFC and ALC, some GO terms unique to each cell type were identified. Specifically, GO terms related to adhesion were only reported for MFC and included *thrombospondin-1* (*TSP-1*), a well known cell adhesion glycoprotein that mediates cell-to-cell and cell-to-matrix interactions [55]. Conversely, *TSP-1* was involved in phosphate metabolic processes for ALC. TSP-1 is a multifunctional protein that plays a role in a variety of biological activities including inhibition of angiogenesis, regulation of cell proliferation, inflammation and wound healing and phosphorylation [56–58]. Similarly, the GO term “cellular homeostasis” was only identified in MSC, a heterogeneous population of stem and progenitor cells that is essential to skeletal muscle embryonic development, repair, homeostasis and senescence [59]. Decreased satellite cell homeostasis has been linked to several muscular disorders [8], and pathway analysis further supported the functional analysis data, indicating processes involved in cytoskeleton, proteoglycans and cell adhesion play a key role in differentiation. Pathway analysis also confirmed that signaling pathways and carbohydrate metabolic pathways are the main pathways altered in differentiation. Further studies of genes annotated in these pathways may reveal their novel role during myogenesis.

C2C12 murine myoblasts cell line was used to validate our bovine EST data since they are considered a model system for the study of skeletal muscle development [60]. The bovine MSC primary cells have certain limiting factors, including loss of their characteristic features with multiple subcultures and fragility during chemical treatment in knockdown experiments; therefore, we limited our functional study to C2C12 cells. Specifically, the expression of *DDAH2* and *S100A4* during MFC formation and *HBA2* and *PTX3* during ALC formation was analyzed. The highest EST numbers were the main criteria for selecting these genes (*DDAH2* and *HBA2*). *DDAH2* has 20 ESTs representing MFC, while MSCs and ALCs had only 1 and 0 ESTs respectively. Similarly, *HBA2* had 15 ESTs in ALCs and none in MSCs and MFCs. Two additional genes that have been reported elsewhere, *PTX3* and *S100A4* [61,62] were validated in the current study. Real time RT-PCR revealed that *DDAH2* and*S100A4*exhibited the maximum level of mRNA expression at Day 2 at the time of cell alignment before myotube formation. S100A4 is a calcium binding protein that exerts its effects by interacting with and modulating activity of other proteins [63]. In addition to our study of detection of *S100A4* mRNA in myoblast and myotube formed cells, S100A4 protein has been reported in rat myocytes [64]. It has also been suggested that *S100A4* modulates cell shape and motility by interacting with components of the cytoskeleton [65], an important biological process during MFC formation involving many genes as indicated by the functional and pathway analysis above. Increased DDAH2 protein expression during myogenesis and slightly distorted cell morphology, reduced cell alignment, and decreased *MYOG* mRNA expression after *DDAH2* knockdown indicated its definite role during myogenesis. *DDAH2* is expressed in tissues expressing endothelial NOS (eNOS) [66] and is involved in nitric oxide (NO) production [67]. NO triggers signaling pathways involved in myogenesis [37,68]; thus, it is reasonable to speculate that modulation of NO is required during skeletal myogenesis. 

Hemoglobin comprising of a heme and globin is one of the best characterized proteins and a carrier of oxygen in red blood cells [69]. Hemoglobin is composed of two alpha and two beta chains encoded by different genes located on different chromosomes. Two types of alpha chains that form *HBA1* and *HBA2* share sequence similarity in the ORF, but differ by a few amino acids in the 5’ and 3’ region [70,71]. However, the ESTs obtained from our study revealed 100% homology with *HBA2* of *Bos Taurus*. Detection of *HBA2* mRNA expression in skeletal muscle by microarray analysis is in agreement with the results reported by Raymond et al. [72]. Here, we describe a transient increase of *HBA2*mRNAlevel followed by its protein expression during transdifferentiation of C2C12 myoblast cells into ALCs. However, we were not able to detect expression of the *HBB* gene upon real time RT-PCR analysis. Indeed, neither of the PCR primer sets, including the one described previously by other researchers [73], produced the expected amplicon (data not shown). A similar study revealed both mRNA and protein expression of *HBA2* in endothelial cells, but no expression of *HBB* [74]. Further, when a knockdown experiment was carried out to elucidate the role of *HBA2* during transdifferentiation of MSCs into ALCs, a decrease in intracellular fat accumulation was observed. Decreased mRNA expression of lipid transporters (*CD36* and *FABP4*) after *HBA2* knockdown is in accordance with low intracellular fat accumulation. *CD36* and *FABP4* are well known markers of adipogenesis [75] that play significant roles in NO signaling [76,77]. It was also recently shown that endothelial cell expression of haemoglobin α regulates nitric oxide signalling [74]. Moreover, when checked in 3T3-L1 preadipocytes, the *HBA2* gene showed high up-regulation during adipogenesis (data not shown), indicating a definite common role of *HBA2* in terms of lipid accumulation during transdifferentiation of MSCs into adipocytes and differentiation of preadipocytes into adipocytes. Since we failed to detect the *HBB* expression in both C2C12 and 3T3-L1 cells, it is still unclear whether *HBA2* modulates intracellular lipid accumulation during adipogenesis with or without *HBB*. 

Overall, this study is an attempt to identify key genes responsible for MFC formation and ALC formation from MSC. Our observations illustrate that knockdown of *DDAH2* and *HBA2* perturbs genes involved in nitric oxide signaling. An apparent role of *HBA2* in ALC formation is described here for the first time. Additional investigation of the genes identified in this study will help elucidate the mechanism responsible for MFC and ALC formation. 

## Supporting Information

Figure S1 **MSCs differentiation and transdifferentiation.** MSCs was isolated from bovine hind leg muscles and cultured for 10 days (A). MSCs grown in DMEM +10% FBS +1% P/S for 14 days formed MFC (B) and in TDM for 7 days formed ALC (C).
(TIF)

Figure S2 **MyoD expression in MSCs.** Cellular localization of MyoD by immunocytochemistry in bovine MSCs. (A) Cell picture at Day11. (B) DAPI-stained nuclei. (C) MyoD antibody stained cells.
(TIF)

Table S1 **Primer information.**
(XLS)

Table S2 **The genes in this list consist of five or more ESTs and were used for DAVID functional analysis.** The lists of highly expressed genes analyzed by DAVID are provided as separate sheets for MSC, MFC and ALC.
(XLSX)

Table S3 **Complete list of statistically significant (p-value ≤ 0.01) GO terms generated by DAVID functional analysis.**
(XLSX)

Table S4 **List of all KEGG pathways reported for highly expressed gene lists.**
(XLSX)
