# Supplementary figures and images for: Expressed Sequence Tags for Bovine Muscle Satellite Cells, Myotube Formed-Cells and Adipocyte-Like Cells
Source: PLoS One. 2013 Nov 5;8(11):e79780. doi: 10.1371/journal.pone.0079780 (PMC3818215; doi:10.1371/journal.pone.0079780)

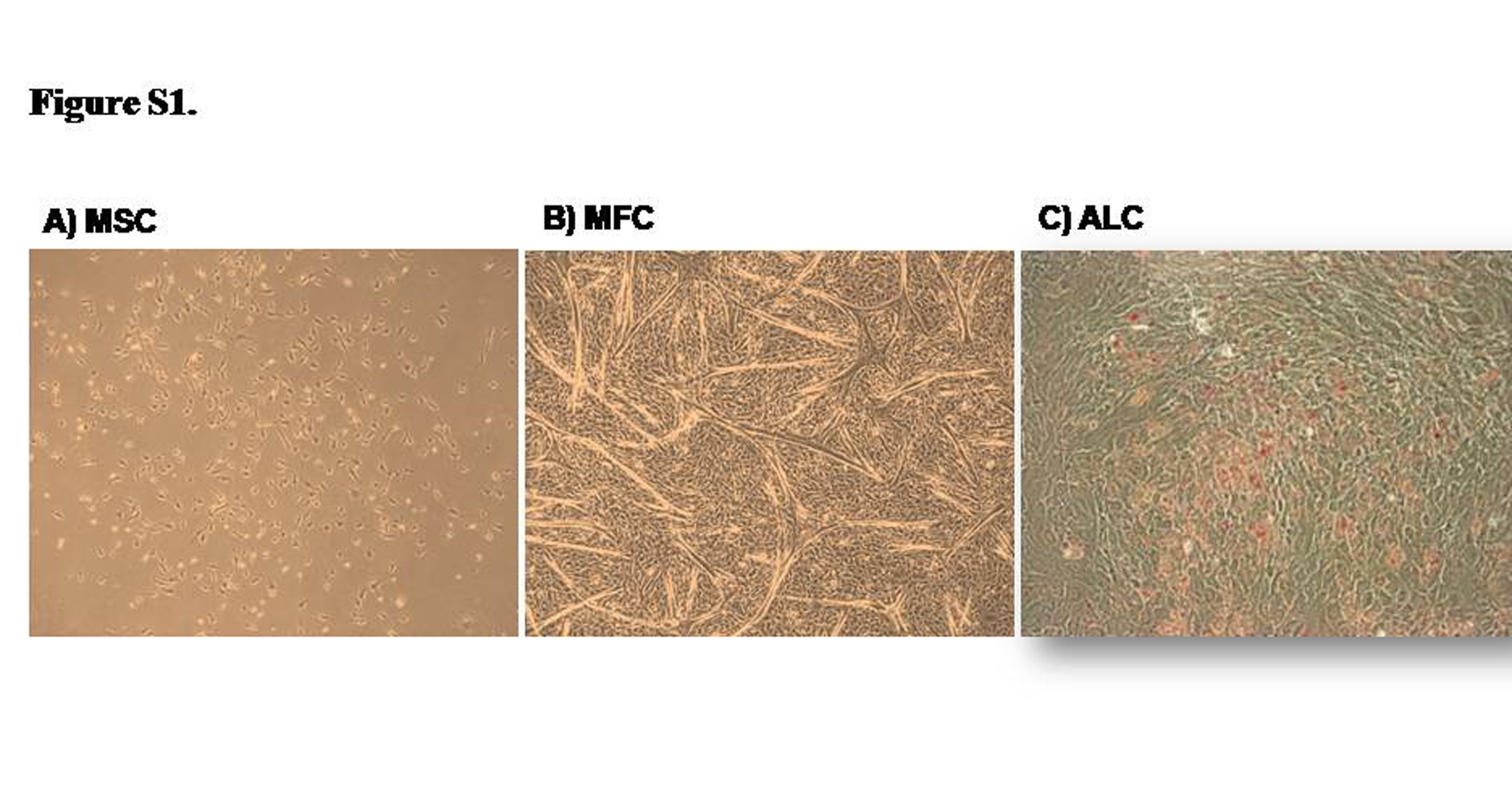

Supplement: Figure S1 — MSCs differentiation and transdifferentiation. MSCs was isolated from bovine hind leg muscles and cultured for 10 days (A). MSCs grown in DMEM +10% FBS +1% P/S for 14 days formed MFC (B) and in TDM for 7 days formed ALC (C). (TIF) [file pone.0079780.s001.tif]

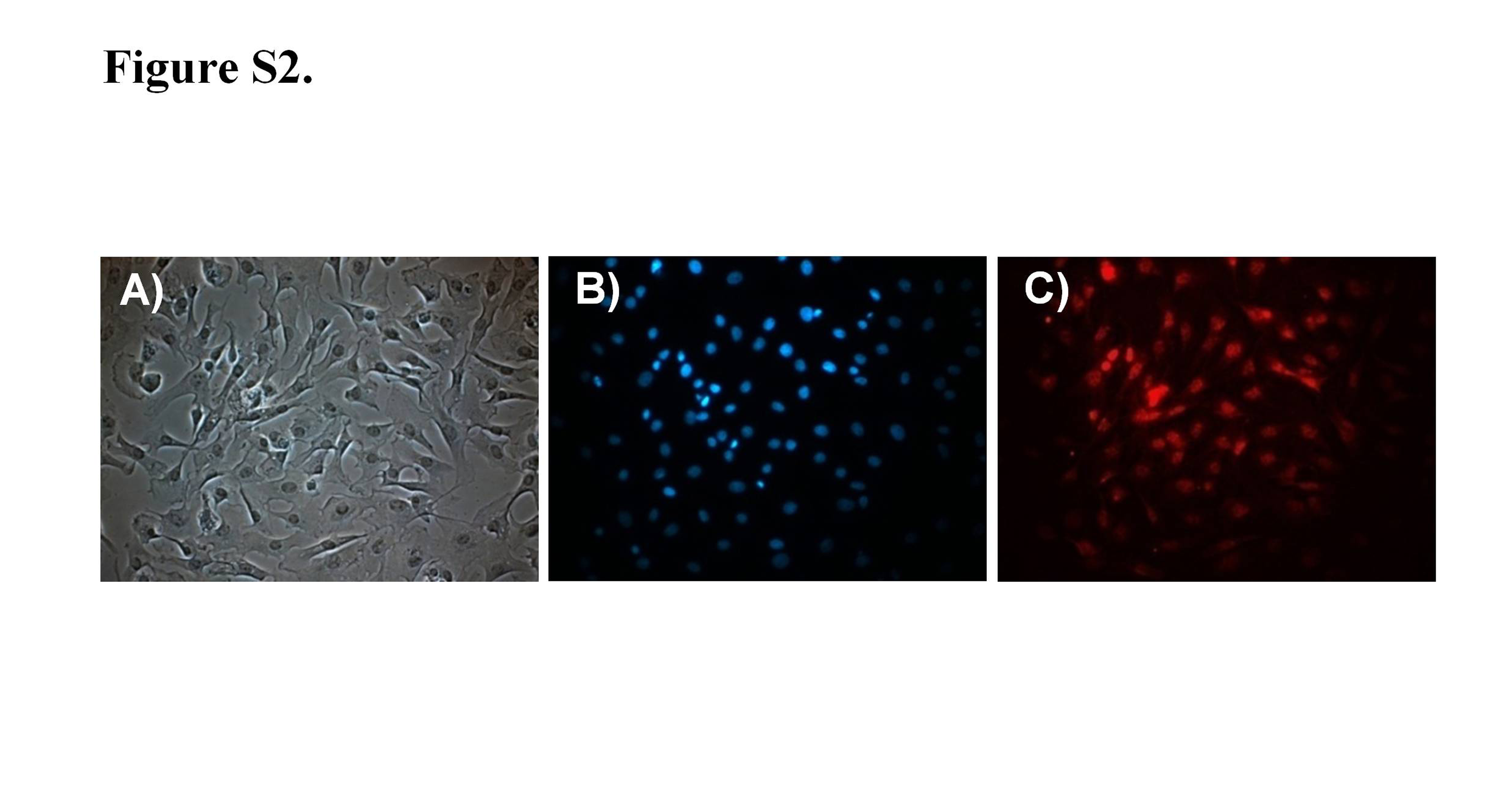

Supplement: Figure S2 — MyoD expression in MSCs. Cellular localization of MyoD by immunocytochemistry in bovine MSCs. (A) Cell picture at Day11. (B) DAPI-stained nuclei. (C) MyoD antibody stained cells. (TIF) [file pone.0079780.s002.tif]
